# MOZ-TIF2 repression of nuclear receptor-mediated transcription requires multiple domains in MOZ and in the CID domain of TIF2

**DOI:** 10.1186/1476-4598-6-51

**Published:** 2007-08-13

**Authors:** Hong Yin, Jonathan Glass, Kerry L Blanchard

**Affiliations:** 1 Feist-Weiller Cancer Center and Department of Medicine, Louisiana State University Health Sciences Center, Shreveport, USA; 2 Eli Lilly & Company, Indianapolis, USA

## Abstract

**Background:**

Fusion of the MOZ and TIF2 genes by an inv (8) (p11q13) translocation has been identified in patients with acute mixed-lineage leukemia. Characterization of the molecular structure of the MOZ-TIF2 fusion protein suggested that the fusion protein would effect on nuclear receptor signaling.

**Results:**

A series of deletions from the N-terminus of the MOZ-TIF2 fusion protein demonstrated that the MOZ portion is essential for nuclear localization of the fusion protein. Transient expression of MOZ-TIF2 dramatically decreased both basal and estradiol inducible reporter gene activity in an estrogen receptor element (ERE) driven luciferase reporter system and decreased androgen-inducible reporter gene activity in an androgen receptor element (ARE) luciferase reporter system. Deletions in the MOZ portion of the MOZ-TIF2 fusion protein reduced the suppression in the ER reporter system. Stable expression of MOZ-TIF2 inhibited retinoic acid (RA) inducible endogenous CD11b and C/EBPβ gene response. The suppression of the reporter systems was released with either a CID domain deletion or with mutations of leucine-rich repeats in the TIF2 portion of MOZ-TIF2. The co-expression of TIF2, but not CBP, with MOZ-TIF2 partially restored the inhibition of the reporter systems. In addition, analysis of protein interactions demonstrated MOZ-TIF2 interaction with the C-terminus of CBP through both the MOZ and TIF2 portions of the fusion protein.

**Conclusion:**

MOZ-TIF2 inhibited nuclear receptor-mediated gene response by aberrant recruitment of CBP and both the MOZ and TIF2 portions are required for this inhibition.

## Background

Chromosomal translocations resulting in MOZ-(monocytic leukemia zinc finger protein)-TIF2 (transcriptional intermediary factor 2) fusions occur in acute myelogenous leukemia and most commonly have been seen with AML of the French-American-British phenotype of M4/M5 subtype. The MOZ-TIF2 fusion protein consists of the N-terminus of MOZ and the C-terminus of TIF2. Patients with these translocations often exhibit rapid progression and poor response to therapy. Various translocations involving MOZ have been described such as MOZ-CBP (cAMP-response element binding protein t(8;16)(p11;p13), MOZ-P300 t(8;22)(p11;q13), and MOZ-TIF2 (inv(8)(p11q13). In a pediatric patient with therapy-related myelodysplastic syndrome a MOZ translocation was found between t(2;8)(p23;p11) [1-8]. In addition, a MOZ homologous protein, MORF (monocytic leukemia zinc finger protein-related factor) has been found fused to CBP via t(10;16)(q22;p13) in patients with AML and therapy-related myelodysplastic syndromes [9-11].

MOZ is a histone acetyltransferase (HAT) [12,13] and plays a role in maintenance of hematopoietic stem cells [14,15]. MOZ also functions as a transcription regulator to activate RUNX1 and RUNX2-mediated transcription through protein-protein interactions. Co-expression of RUNX1 and MOZ can synergistically activate the MIP-1 alpha promoter through a proximal RUNX binding site [16,17]. The N- and C-termini of MOZ have different functions with the N-terminus responsible for transcription repression and the C-terminus for transcription activation. The MOZ-CBP fusion blocks RUNX1-mediated transcription. We have previously identified by yeast two-hybrid analysis and co-immunoprecipitation two human chromatin assembly factors, the p150 subunit of chromatin assembly factor (CAF) and anti-silencing function 1b (ASF1b), that interact with MOZ and the MOZ portion of the MOZ-TIF2 protein [18]. In zebrafish, MOZ through its crucial histone acetyltransferase activity regulated Hox expression. A MOZ mutation caused a late defect in facial motor-neuron migration and led to a abnormality in pharyngeal arch developmental [19].

TIF2 belongs to the p160 protein family which also includes SRC-1 (Steroid receptor coactivator), TIF2/GRIP1/SRC-2, and pCIP/ACTR/AIB-1/RAC-3/TRAM-1/SRC-3. The functions of the p160 family have been well reviewed [20-24]. The molecular structure of TIF2 demonstrates several functional domains including a PAS/bHLH domain, a receptor interaction region, and two activation domains (AD) [25-28]. In nuclear receptor signaling, TIF2 binds to nuclear receptors predominately through its nuclear receptor interacting domain (NID) [29,30] and recruits the transcriptional co-activators CBP/p300 through CBP interaction domain (CID/AD1) [27,31] and CARM-1, an arginine methytransferase, through AD2 [32-35]. As a consequence, acetylation and methylation in histone H3 and the KIX domain of CBP/p300 activates the promoter and facilitates the basal transcriptional machinery. TIF2 knock-out mice displayed significantly reduced fertility and abnormalities in white adipose tissue and energy metabolism [36-38].

The expression of MOZ-TIF2 in a mouse model resulted in acute myelogenous leukemia (AML) and blocked the differentiation of stem cells to hematopoietic progenitors [39,40]. The deletion of the CID in the TIF2 partner of MOZ-TIF2 abolished the leukemogenesis and blocked the inhibition by MOZ-TIF2 of RAR, PPAR, and p53-mediated transcription [41]. MOZ-TIF2 also altered cofactor recruitment and histone modification at the RARbeta2 promoter [42]. In this study, we demonstrate that the MOZ portion of the MOZ-TIF2 fusion protein is essential for nuclear localization of MOZ-TIF2 and describe MOZ-TIF2 repression of transcriptional activation by ER and AR. This inhibition depended not only on the CID domain in TIF2 portion but also on multiple domains in the MOZ portion. The forced expression of TIF2, but not CBP, could reverse the inhibition. Stable expression of MOZ-TIF2 altered the retinoid acid (RA)-mediated endogenous gene responses.

## Results

### The structure of MOZ-TIF2 and expression of the fusion gene

**Figure 1:**
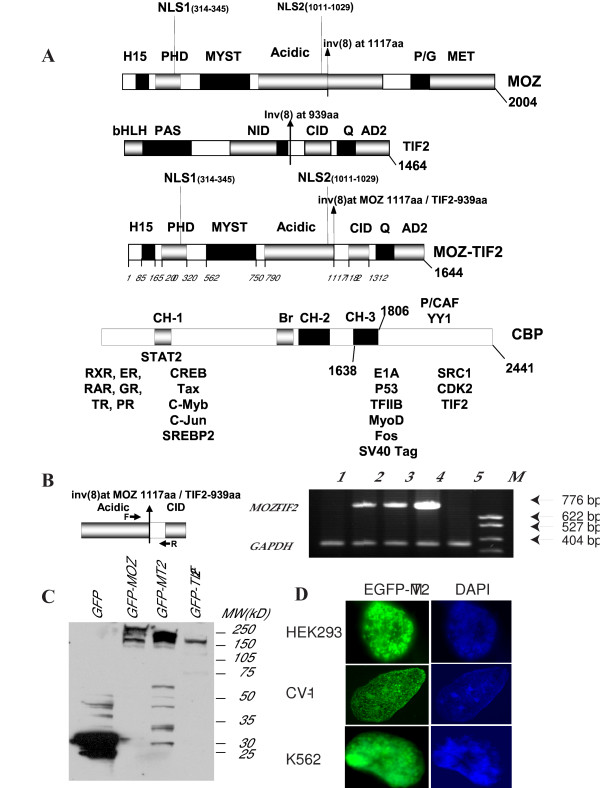
**Schematic structure of the MOZ-TIF2 fusion gene and expression and localization of MOZ-TIF2**. **A. **Schematic representation of human MOZ, human TIF2, the MOZ-TIF2 fusion protein, and CBP. The break point for MOZ is amino acid 1117 and for TIF2 is amino acid 939. **B. **Expression of MOZ-TIF2 RNA. Left panel, schematic representation of PCR primers (thick arrows) in fusion region of MOZ-TIF2. F, forward primer and R, reverse primer. Right panel, RNA expression of MOZ-TIF2 as detected by RT-PCR in transduced or transfected cells. Lane 1, NIH-3T3 cells transduced with retrovirus control. Lane 2, NIH-3T3 cells transduced with a retrovirus expressing MOZ-TIF2 fusion protein. Lane 3 and Lane 4, HEK 293 cells (Lane 3) and K562 cells (Lane 4) transfected with pcDNA3.1-MOZ-TIF2. Lane 5, K562 cells transfected with pcDNA3.1 alone. Lane M, molecular weight markers. **C. **Expression of EGFP-tagged MOZ, MOZ-TIF2, and TIF2 proteins in HEK293 cells. Cells were transiently transfected with various pEGFP fusion constructs, cell lysates extracted 36 hours later and separated by SDS-PAGE (4–20% polyacrylamide). Western blot analysis was performed to detect EGFP tagged proteins of MOZ, MOZ-TIF2, and TIF2 with mouse monoclonal antibody to EGFP. MW, molecule markers in kilodaltons (kD). **D. **The localization of EGFP-tagged MOZ-TIF2 in HEK 293, CV-1, and K562 cells with the nuclei stained with DAPI.

**Figure 2:**
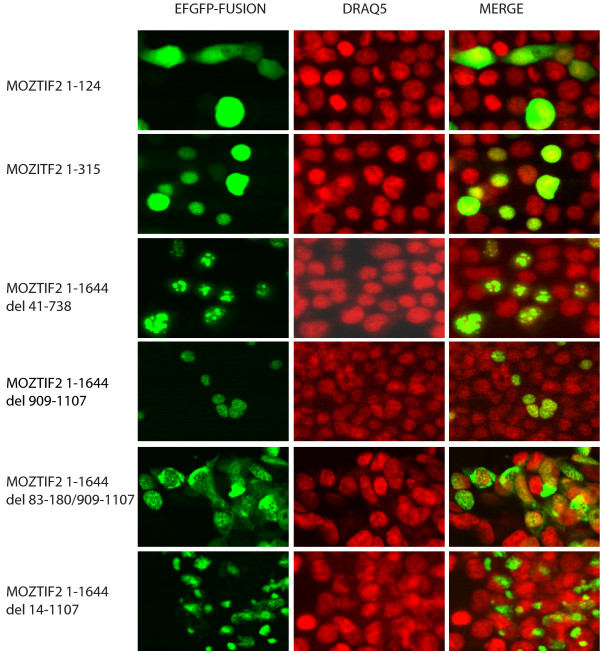
**The MOZ portion of MOZ-TIF2 is essential for nuclear localization of MOZ-TIF2**. EGFP fusions with MOZ-TIF2 fragments were transiently transfected into HEK 293 cells. After 24 hour, localization of EGFP fusion protein in HEK 293 cells was observed by confocal microscopy. Nuclei were stained in live HEK 293 cells with DRAQ5™ (AXXORA, LLC, San Diego, CA).

More than 5 cases of AML of the M4/M5 subtype have been reported with chromosome translocation of inv (8) (p11q13). The fusion in the patient at our institution occurred at nucleotide position 3744 in the MOZ coding region and nucleotide position 2974 in the TIF2 coding region. The schematic structure of MOZ-TIF2 is shown in Figure 1A. The fusion protein contains from the MOZ moiety the PHD zinc finger domain, the MYST domain, and a region rich in acidic amino acids and from the TIF2 portion the CID domain, the Q-rich region, and the AD2 domain. After transient transfection with the MOZ-TIF2 expression plasmid or transduction with a retrovirus expressing the MOZ-TIF2 fusion (Figure 1B) expression of MOZ-TIF2 RNA was seen in HEK 293, K562, and NIH3T3 cells. Using an EGFP-tagged MOZ-TIF2 construct expression of the fusion protein was detected by western analysis (Figure 1C) and could be seen localized to the nuclei of transfected cells (Figure 1D). Examination of the protein sequence of the fusion protein suggests that in MOZ-TIF2 there are two potential nuclear localization signals located between amino acids 83–180 and 909–1107. However, MOZ-TIF2 with deletion of either amino acid 41–738 (Figure 2, third row) or 909–1107 (Figure 2, fourth row) still localized to the nucleus. Even deletion of both potential nuclear localization regions could not exclude the MOZ-TIF2 completely from the nucleus (Figure 2, fifth row). Only deletion of a considerable amount of the MOZ portion caused the protein localization to shift from nucleus to cytoplasm (Figure 2, sixth row).

### MOZ-TIF2 affects the transcription activation of ER and AR

**Figure 3:**
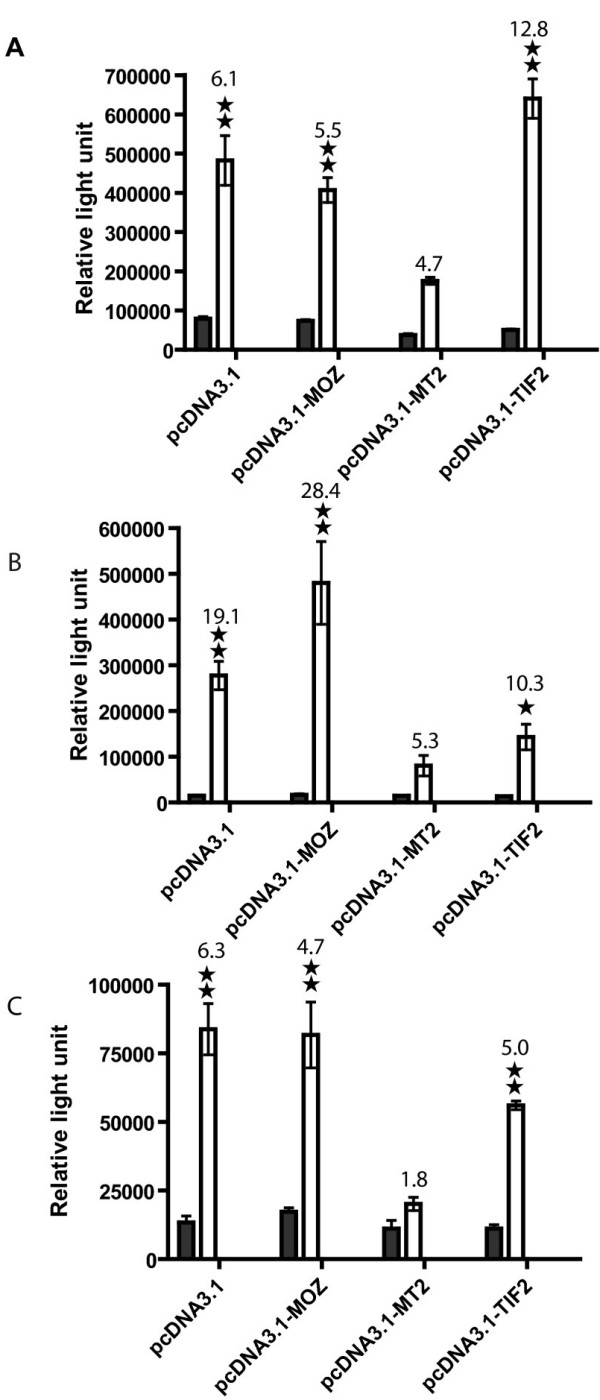
**MOZ-TIF2 inhibits the transcriptional activation of ER and AR**. **A. **MOZ-TIF2 (MT2) and ER activity. pVit-TKSL, an estrogen response element-driven luciferase reporter plasmid was co-transfected into CV-1 cells with an estrogen receptor expression vector and pCDNA3.1-MOZ, pCDNA3.1-MOZ-TIF2 (MT2), pCDNA3.1-TIF2 or vector alone. **B **and **C**. MOZ-TIF2 and AR activity. Two androgen response element-driven luciferase reporter systems were employed. **B **shows the effect of a plasmid that contains a full length PSA promoter (PL) with multiple androgen response element sites and **C **shows the effect of a plasmid containing a minimum PSA promoter (PC) with only two androgen response elements. Both ARE-containing reporter plasmids were co-transfected with an androgen receptor expression vector and either pCDNA3.1-MOZ, pCDNA3.1-MOZTIF2 (MT2), pCDNA3.1-TIF2 or vector alone. 5α-dihydrotestosterone (DHT) was added to a final concentration of 50 nM, the cells collected and luciferase activity measured as described above. Double or single stars represent a significant difference at P < 0.01 or P < 0.05 level respectively by the two tail Student T- test compared to the transfection with MOZ-TIF2 in the ligand added condition. Open bars, 50 nM estradiol (A) or DHT (B and C). Dark bars, no added estradiol (A) or DHT (B and C). The numbers on top of the open bars, i.e. added ligand, are the ratios of light units in presence of the ligand to light units in the absence of ligand. The percentage inhibition noted in the text was calculated from the percent of light units resulting from induction by estrogen in MOZ-TIF2 expressing cells compared to pcDNA-transfected control cells subtracted from 100%.

TIF2 is a co-activator of nuclear hormone receptors and its two activation domains, CID and AD2, are retained in the MOZ-TIF2 fusion. To determine if the MOZ-TIF2 fusion protein alters the co-activator function of wild type TIF2 in nuclear receptor mediated transcription activation we used a luciferase reporter system driven either by a promoter containing the estrogen receptor binding element (ERE) or the androgen receptor binding element (ARE). Co-transfection of the luciferase reporter ERE plasmid with MOZ-TIF2 into CV-1 cells caused a significant decrease in luciferase activity both in basal and estrogen induced conditions (Figure 3A) compared to the co-transfection with wild type MOZ, TIF2, or vector alone. The induction by estrogen in MOZ-TIF2 was inhibited by 64 % compared to that in pcDNA3 control. The inhibition of AR-activated transcription by MOZ-TIF2 was also demonstrated with the full length PSA promoter/enhancer (PL) that has multiple AREs and with a short promoter (PC) that has two AREs. Compared to MOZ, TIF2, and vector alone, there was a significant suppression by MOZ-TIF2 either with the PL PSA promoter (Figure 3B) or the PC PSA promoter (Figure 3C).

### The leucine-rich repeats in the CID of TIF2 determines the inhibition in transcription activation of ER and AR by MOZ-TIF2

**Figure 4:**
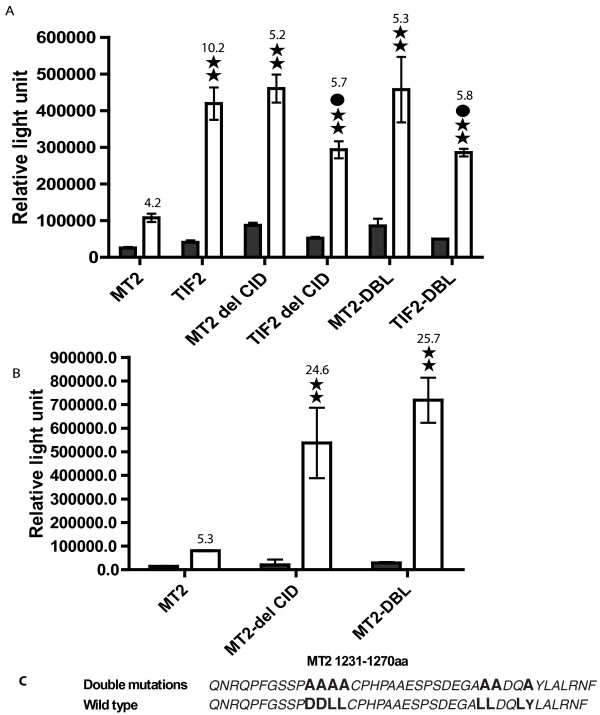
**Deletion of the CID domain and mutations in two leucine-rich repeats in the CID domain of the TIF2 portion of MOZ-TIF2 released the inhibitory effect of MOZ-TIF2 on the transcriptional activation of ER and AR in CV-1 cells**. **A**. ER signaling. pVit-TKSL was co-transfected with an estrogen receptor expression plasmid (hER) into CV-1 cells. **B**. AR signaling. The reporter plasmid PL was used as described in Figure 3. Del CID: The CID domain was deleted from MOZ-TIF2 (MT2 delCID) (amino acids 1182–1312) and TIF2 (TF2 delCID) (amino acids 1002–1132). DBL : mutations were created in the leucine-repeat regions of CID of MOZ-TIF2 (MT2DBL) and TIF2 (TF2 DBL) as shown in panel **C**. The letters in bold type in C represent the mutated amino acids. Open bars, 50 nM estradiol (A) or DHT (B). Dark bars, no added estradiol (A) or DHT (B). Double stars represent a significant difference at P < 0.01 by the two-tailed Student T- test compared to the transfection with MOZ-TIF2 in the ligand added condition. Solid circles represent a significant difference at P < 0.05 by two-tailed Student T- test compared to the transfection with TIF2 in the ligand added condition. The numbers on top of the open bars indicate the ratio of light units in the presence and absence of ligand as described in Figure 3. The fold increase given in the text was calculated as light units with the mutated MOZ-TIF2 compared to the wild type MOZ-TIF2 in absence or presence of ligand and fold decrease was calculated as the light units with the wild type TIF2 compared to the light units with the CID deleted TIF2.

The CID domain of the TIF2 protein is important to recruit CBP/p300 into the nuclear receptor mediated transcriptional complex to acetylate histone and non-histone proteins and to promote transcription. However, as shown above MOZ-TIF2 exhibited decreased ER and AR-mediated transcription. To determine if altered recruitment of CBP/p300 into the nuclear receptor mediated transcriptional complex is responsible for inhibition of transcription of the MOZ-TIF2 fusion protein, we examined the effects of deletion of the CID domain both in the intact TIF2 as well as the MOZ-TIF2 fusion protein (Figure 4). Compared with the wild type MOZ-TIF2, the MOZ-TIF2 with deleted CID exhibited increased transcription activation of ER (Figure 4A) by 3.4 and 4.3 fold under both basal and estrogen stimulated conditions, respectively. In contrast, the deletion of CID in TIF2 reduced estrogen induction by 1.4 fold compared to wild type TIF2 (Figure 4A). With AR-mediated transcription, the CID deletion increased androgen induction by 6.7 fold compared to intact MOZ-TIF2 (Figure 4B). These results suggest that the CID in MOZ-TIF2 is necessary for inhibition of ER and AR-mediated transcription activation and has an opposite effect in the wild type TIF2.

We next mutated the two leucine-rich repeats PDDLL and LLDQL in the CID domain of MOZ-TIF2 by mutating several leucines to alanines in the region between amino acids 1231 and 1270 (Figure 4C). The LLDQL repeat has previously been demonstrated to be a CBP binding motif [27] The mutation of two repeats significantly stimulated both estrogen (Figure 4A) and androgen (Figure 4B), activated transcription compared to wild type MOZ-TIF2. With the mutated MOZ-TIF2, ligand-stimulated transcription increased approximately 4.2 fold for ER and 8.9 fold for AR, respectively.

### MOZ-TIF2-mediated repression of transcription can be partially restored by TIF2 but not CBP

**Figure 5:**
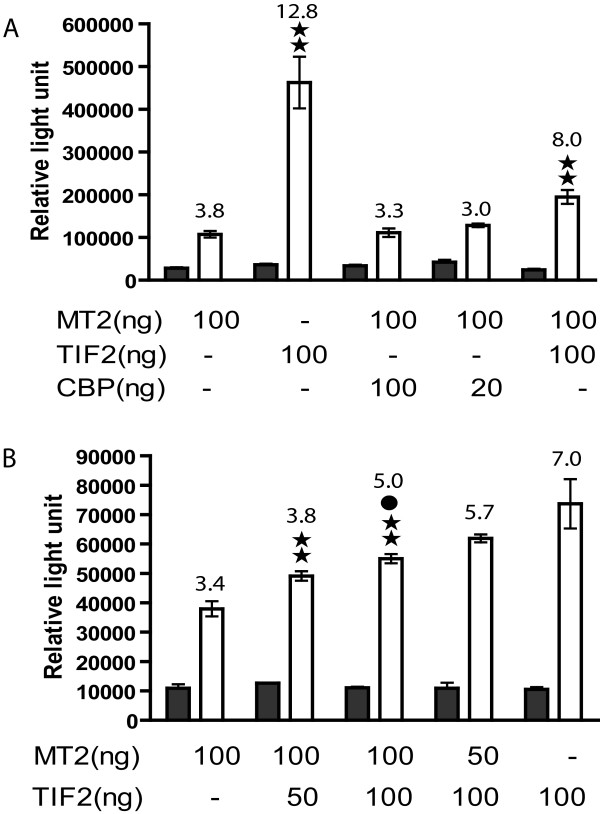
**MOZ-TIF2 inhibition of ER-mediated transcription was not antagonized by increased expression of CBP and expression of MOZ-TIF2 competed with wild type TIF2 in ER signaling**. CV-1 cells were co-transfected with pVit-TKSL containing MOZ-TIF2 or MOZ-TIF2 plus CBP or TIF2. The cells were lysed and luciferase activity was measured as described after incubation of the transfected cells for 36 hours of cells with 50 nM estradiol. **A**. The forced expression of CBP does not antagonize MOZ-TIF2 inhibition of ER-mediated transcription. **B**. The inhibition of ER-mediated transcription by MOZ-TIF2 was partially released by increased expression of wild type of TIF2. Open bars, the presence of 50 nM estradiol; dark bars, without estradiol. Double stars represent a significant difference at P < 0.01 by the two tailed Student T- test compared to the transfection with MOZ-TIF2 in the ligand added condition. Dark circles represent a significant difference at P < 0.05 compared to the transfection with TIF2 alone in the ligand added condition. The numbers on top of the open bars indicate the ratio of light units as described in Figure 3.

**Figure 6:**
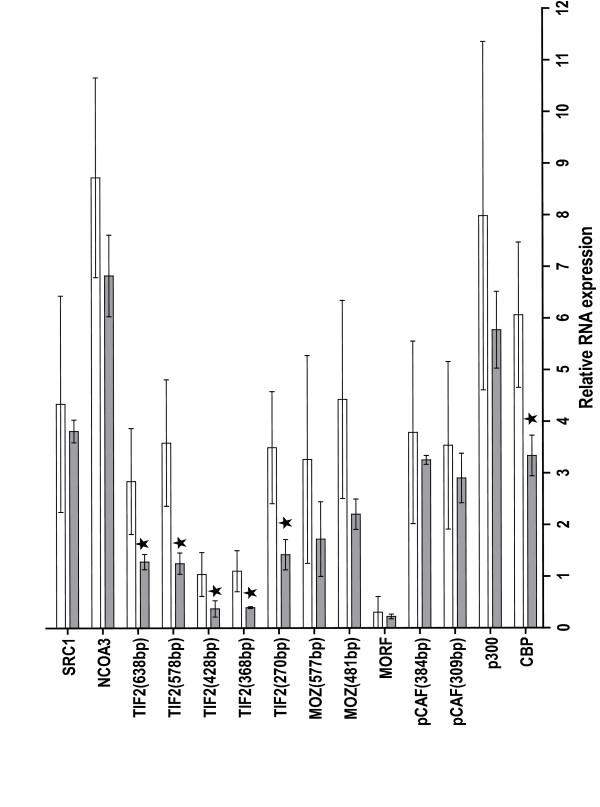
**RNA expression in leukemic blasts from AML patients with or without MOZ-TIF2 fusion**. RNA was isolated from the leukemic blasts of 5 different patients. RNA was also isolated on three separate occasions from the leukemic blasts of the patient exhibiting the MOZ-TIF2 fusion. RT-PCR was conducted as described in Experimental Procedures using the primers listed in Table 1. Shown are the results as relative amounts of RNA in the non-MOZ-TIF2 leukemic blasts (clear bars) compared to the MOZ-TIF2 blasts (shaded bars). Stars represent a significant difference at P < 0.05 by the two-tailed Student T-test comparing RNA levels in the blasts of the patients without the MOZ-TIF2 fusion to the levels in the blasts with the MOZ-TIF2 fusion.

CBP participates in many transcription events and may, therefore, be simultaneously required by multiple pathways. An example of competition for CBP has been shown for the hematopoietic Ets transcription factor Spi-B competing for CBP with c-Myb [43]. In addition, forced expression of CBP in a p53 deficient cell line, SaOS-2, released the inhibition of p53-mediated transcription by MOZ-TIF2 [41]. To determine if CBP was a limiting factor leading to inhibition by MOZ-TIF2 of the transcriptional activation of the ER or AR reporter constructs, MOZ-TIF2 was co-transfected with a plasmid expressing CBP. However, forced expression of CBP did not reverse the MOZ-TIF2-mediated suppression of ER mediated transcription (Figure 5A) nor of AR-mediated transcription (data not shown), suggesting that CBP was not a limiting factor in our experimental systems. In contrast, co-transfection of TIF2 with MOZ-TIF2 reversed the inhibition of ER-mediated transcription by MOZ-TIF2 (Figure 5B). Hence, the relative deficiency in transcription co-factors contributes to the inhibition of nuclear receptor-mediated transcription by MOZ-TIF2. To address if the levels of transcription cofactors could be limiting *in vivo*, we examined the RNA expression of several transcription co-factors in leukemic blasts and found significant decreases in RNA expression of TIF2 and CBP in the cells of the patient with the MOZ-TIF2 fusion compared to levels in leukemic blasts from patients without the MOZ-TIF2 fusion (Figure 6).

### MOZ-TIF2 interacts with CBP in the ER-mediated transcription complex

**Figure 7:**
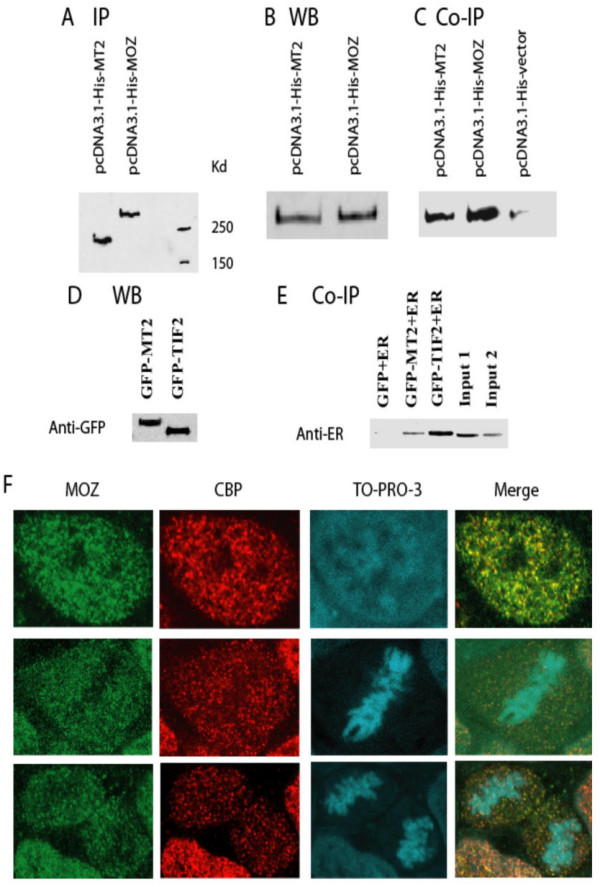
**MOZ-TIF2 participates in the ER activation complex as does TIF2 and interacts with CBP in *vivo***. **A, B, and C. **For identification of the interaction between MOZ-TIF2 and CBP, HEK 293 cells were transiently transfected with His-tagged MOZ, MOZ-TIF2, and vector alone. After 36 hours, the cells were lysed and co-immunoprecipitation conducted by an anti His-tag antibody conjugated to agarose beads. The co-immunoprecipitated proteins were separated by SDS-PAGE and western analyses were performed with an anti-CBP antibody. The input represents 5% of the protein used in the co-immunoprecipitation and was from cells transfected with MOZ-TIF2. **A**. Western blot showing MOZ and MOZ-TIF2 bound to the agarose beads. **B**. Transfected cells subjected to SDS-PAGE to demonstrate the presence of CBP in the transfected HEK293 cells. **C**. Western blot analysis to demonstrate CBP co-immunoprecipitating with MOZ and MOZ-TIF2. **D **and **E. **To determine the presence of MOZ-TIF2 in ER activation complexes, HEK 293 cells were transiently co-transfected with pEGFP-MOZ-TIF2 or pEGFP-TIF2 and with an expression plasmid for the estrogen receptor, RSV-hER. After the addition of 50 nM estradiol for 36 hours, the cells were lysed and co-immunoprecipitation was performed with 500 μg protein using an anti-EGFP antibody. The co-immunoprecipitated proteins were subjected to 12% SDS-PAGE fractionation and ER was detected by western blot with anti-ER antibody. The input represents 5% of the protein used in the co-immunoprecipitation. **D**. The expression level of EGFP fusions in cells transfected with MOZ-TIF2 (GFP-MT2) and TIF2 (GFP-TIF2). **E**. The co-immunoprecipitation of ER by MOZ-TIF2 or TIF2. Input 1 is from cells transfected with pEGFP-TIF2 and input 2 is from cells transfected with pEGFP-MOZ-TIF2. **F**. Co-localization between endogenous MOZ and CBP in HEK293 cells during the cell cycle. Top panel, co-localization of MOZ and CBP in the nucleus during G1-S-G2 stage. Middle panel, the co-localization disassociated from chromosomes during the metaphase. Bottom panel, the restoration of co-localization with chromatin.

The above results suggest that an interaction between MOZ-TIF2 and CBP could be essential for the inhibition of ER and AR-mediated transcription by MOZ-TIF2. To obtain direct evidence for the recruitment of CBP by the MOZ-TIF2 fusion protein HEK293 cells were transfected with His-tag MOZ and MOZ-TIF2 (Figure 7A, B, and 7C). In these experiments CBP was co-precipitated both by MOZ-TIF2 and interestingly by MOZ, too (Figure 7C). Corrected for the expression of CBP (Figure 7B) and precipitated MOZ and MOZ-TIF2 (Figure 7A), the ratio of CBP co-precipitating with MOZ and CBP co-precipitating with MOZ-TIF2 was 2 to 1. Interestingly, p300, a CBP-like histone acetylase was co-precipitated by wild type MOZ and MOZ-TIF2 (data not shown). Furthermore, to demonstrate the participation of MOZ-TIF2 in ER-mediated transcription complex, HEK293 cells were transfected with EGFP-tagged MOZ-TIF2 or TIF2 (Figure 7D). The fusion proteins were then precipitated with anti-EGFP antibody and the co-precipitation of ER was examined by Western blot analysis using anti-ER antibody (Figure 7E). Standardized to the levels of EGFP-MOZ-TIF2 and EGFP-TIF2 (Figure 7A), the ratio of ER co-precipitating with MOZ-TIF2 to ER co-precipitating with TIF2 was 1 to 1.7. As further support for an interaction between MOZ and CBP, we observed the expression and localization of endogenous MOZ and CBP in HEK 293 cells (Figure 7F). MOZ was co-localized with CBP in the nucleus through G1-S-G2 stage (Figure 7F, top panel) but dissociated from the chromosomes in metaphase (Figure 7F, middle panel) only to re-associate with chromatin in cytokinesis at the end of M stage (Figure 7F, bottom panel). A similar pattern of co-localization between MOZ and CBP through the cell cycle could also be demonstrated in HeLa cells (data not shown).

### The MOZ portion of MOZ-TIF2 contributes to CBP binding and nuclear receptor-mediated transcription inhibition

**Figure 8:**
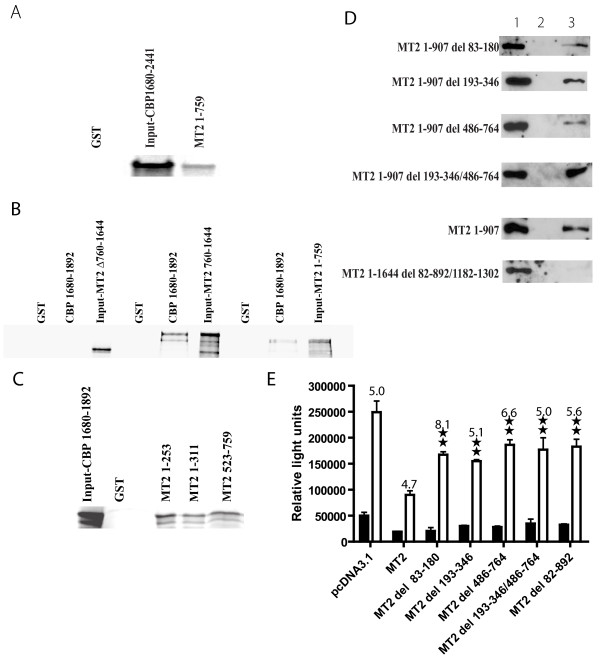
**CBP interacts with multiple sites in the MOZ portion of MOZ-TIF2**. **A**. The MOZ portion (amino acids 1–759) of MOZ-TIF2 interacts with the C-terminus of CBP. As described in Experimental Procedures, a ^35^S-methionine labeled CBP fragment (amino acids 1680–2441) was incubated with the GST-MOZ-TIF2 fragment (amino acids 1–759) (MT2 1–759) and the reaction mixture absorbed to Sepharose 4B-GSH beads. After extensive washing the beads were treated with sample buffer, absorbed proteins separated on SDS-PAGE, and CBP fragments detected with radioautography. GST, the pull-down by GST tag peptide alone mixed with MT2 1–759. CBP 1680–2441, 10 % of the CBP 1680–2441 peptide input into the reaction. MT2 1–759, CBP absorbed to Sepharose-GSH beads through GST-MT2 1–759 peptides. **B**. The MOZ portion of MOZ-TIF2 interacts with the CH3 domain of CBP. Three peptides-MOZ-TIF2 1–759, MOZ-TIF2 760–1644 (amino acids 760–1644), and MOZ-TIF2 760–1644 with deleted CID domain (deleted amino acids 1183–1311)-were labeled with ^35^S-methionine by synthesis by in *vitro *translation. GST-CBP peptides (amino acids 1680–1892) were expressed in *E. coli *and purified by Glutathione Sepharose™ 4B beads. MOZ-TIF2 peptides and GST-CBP peptides were allowed to interact as described in Experimental Procedures and the proteins absorbed to GST-CBP beads were analyzed as described. Input was 10% of ^35^S-methionine labeled polypeptides used in the binding reaction. GST, the pull-down by GST peptide alone mixed with MOZ-TIF2 peptides. CBP 1680–1892, pull-down by GST-CBP peptides (amino acids 1680–1892) incubated with ^35^S-methionine labeled MOZ-TIF2 1–759, MOZ-TIF2 760–1644, and MOZ-TIF2 760–1644 with deleted CID domain. Input-MT2 1–759, Input-MT2 760–1644, and Input-MT2 D 760–1644 are 10 % input of ^35^S-methionine labeled peptides of MOZ-TIF2 1–759, MOZ-TIF2 760–1644, and MOZ-TIF2 760–1644 with deleted CID domain used in the reaction. **C**. The PHD and MYST domains of the MOZ moiety in the fusion protein interact with the CH3 domain of CBP. The ^35^S-methionine labeled CH3 domain peptides of CBP were incubated with GST-MOZ peptides, absorbed to Glutathione Sepharose™ beads, the absorbed proteins separated by SDS-PAGE, and subjected to radioautography. The input represents 10% of ^35^S-methionine labeled peptides used in the binding reaction. GST and input CBP 1680–1892 have the same meanings as described in A above. MT2 1–235, MT2 1–311, and MT2 523–759, peptides of the MOZ partner from amino acids 1–235, 1–311, and 523–759 respectively were used in the incubation with the CH3 domain of CBP. **D**. GST pull-down analysis of CBP binding sites in MOZ partner of MOZ-TIF2. GST tagged C terminal of CBP(amino acids 1680–2441) were expressed in *E. coli *and purified by Glutathione Sepharose™ 4B beads. The expression plasmid with Xpress-tagged MOZ-TIF2 fragments was transfected into HEK 293 cells. After 36 hours whole cell extract was prepared and incubated with GST-tagged C terminal of CBP absorbed onto Glutathione-Sepharose beads. The Xpress-tagged MOZ-TIF2 fragments pulled down by GST-tagged C-terminal of CBP were detected with anti-Xpress antibody and are seen in lane 3. Lane 1 represents 5% of the input of expressed Xpress-tagged MOZ-TIF2 fragment. Lane 2, incubation of Xpress-tagged MOZ-TIF2 fragment with GST peptide alone. **E**. Deletion of CBP binding sites in MOZ portion decreases the inhibition of the transcriptional activation of ER by MOZ-TIF2. As described in Figure 3, an estrogen response element-driven luciferase reporter plasmid was co-transfected into CV-1 cells with an estrogen receptor expression vector and pCDNA3.1 plasmid inserted with MOZ-TIF2 fragments or vector alone. The relative luciferase activity in light units is shown after correction of transfection efficiency by measurement of beta galactosidase activity. Double stars represents a significant difference at P < 0.01 level respectively by two tail student T- test compared to the transfection with MOZ-TIF2 in the ligand added condition. Open bar, 50 nM estradiol. Dark bar, no added estradiol. The numbers on top of the open bars indicate the ratio of light units in the presence and absence of ligand as described in Figure 3.

As CBP co-immunoprecipitated with MOZ and MOZ-TIF2 we now investigated possible interactions of CBP with the MOZ portion of MOZ-TIF2, In these experiments we performed a cell-free GST pull down assay (Figure 8A, B, and 8C) using various fragments of MOZ-TIF2 labeled with ^35^S-methionine by *in vitro *translation. GST fusions with two CBP fragments from the C-terminus of CBP were also synthesized and purified. The CBP C-terminal domain was included in these experiments based on the previous demonstration that the C-terminus was important for the interaction with p53, E1A, and E2F [44,45]. In these assays a ^35^S-methionine labeled peptide consisting of amino acids 1–759 of MOZ-TIF2, was pulled down by the GST tagged C-terminal region of CBP (amino acids 1680 to 2441) (Figure 8A). The binding site of MOZ 1–759 to the C-terminus of CBP was further localized to the CH3 domain of CBP (amino acids 1680 to 1892). The TIF2 portion of MOZ-TIF2 fusion (amino acids 760–1644) also bound to the CH3 domain of CBP but required the CID domain of TIF2 (Figure 8B). Binding between the MOZ portion of MOZ-TIF2 and CBP showed that fragment containing the PHD domain (amino acids 1–253) and a fragment containing the MYST domain (amino acids 523–759) bound to a CBP fragment containing the CH3 domain (amino acids 1860–1892) (Figure 8C). To further demonstrate the multiple binding sites of CBP in MOZ portion, we examined the binding of C-terminal of CBP with region-specific deleted MOZ-TIF2 protein by pull down assay and verified that multiple CBP binding sites existed in the MOZ portion of the MOZ-TIF2 fusion protein (Figure 8D). Deletion of both the PHD and MYST domains (Figure 8D, MT2 1–907 del 193–346/486/764) could not abolish the binding of the MOZ portion fragment to CBP; however, deletion of almost the entire MOZ portion and the CID domain in TIF2 portion blocked the binding of MOZ-TIF2 to CBP (Figure 8D, MT1–1644 del 82–892/1182–1302). To address if multiple CBP binding sites are functionally important, we tested the ER-mediated transcription activity of MOZ-TIF2 proteins with a region specific deletion in MOZ portion (Figure 8E). These results showed that multiple CBP binding sites in the MOZ portion are necessary for MOZ-TIF2 to affect ER transcription and deletion of any of these sites could partially release the transcription inhibition effects seen with MOZ-TIF2.

### MOZ-TIF2 inhibits expression of endogenous RA response genes in U937 cells

**Figure 9:**
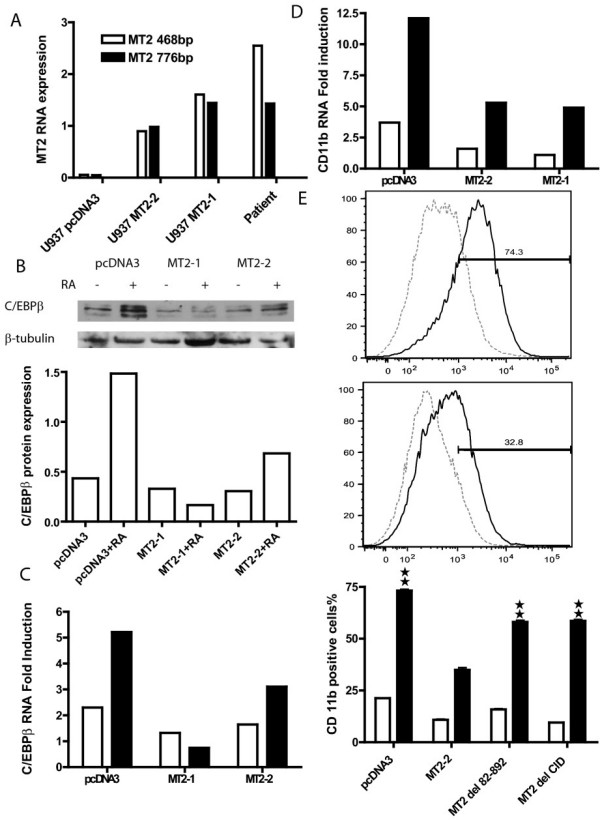
**Stable expression of MOZ-TIF2 in U937 cells down-regulates retinoic acid (RA)-mediated induction of CD11b and C/EBPβ genes**. **A**. Relative expression of MOZ-TIF2 mRNA in stably transfected U937 cells and leukemia patient's blast. U937 cells were transfected with the pcDNA3.1 plasmid carrying the full length MOZ-TIF2 cDNA. After selection with G418, two clones were established, MT2-1 and MT2-2, and expression of MOZ-TIF2 (MT2) mRNA was examined by RT-PCR in the two clones, in the patient's blasts, and in U937 cells stably transfected with vector alone. **B**. Expression of C/EBPβ protein in RA treated U937 cells. Cells were treated with 1 μM RA for 72 hours, whole cell protein extracted with RIPA buffer, and proteins fractionated by SDS-PAGE. Western blot analyses were conducted with anti-C/EBPβ antibody. Top panel, western blot of C/EBPβ and β-tubulin. Bottom panel, relative level of C/EBPβ protein in control and MOZ-TIF2 expressing U937 cells after standardization to β tubulin levels used as a loading control. **C**. Expression of C/EBPβ RNA in RA treated U937 cells. Cells were treated with RA for 24 and 48 hours. RNA was extracted and RT-PCR was conducted as described in Experimental Procedures. The fold-induction by RA was calculated as RA treated sample over non-treated sample for 24 hours (open bars) and 48 hours (dark bars) of RA treatment. **D**. Fold-induction of CD11b RNA in RA treated U937 cells. Cells were treated with 1 μM RA for 8 and 48 hours. RNA was extracted, real-time RT-PCR conducted, and fold-induction by RA calculated comparing the RA treated sample to the non-treated sample. Open and dark bars, 8 and 48 hours of RA treatment respectively. **E**. Percentage of CD11b positive U937 cell after RA treatment. Cells were treated with 1 μM RA for 72 hours and were stained with APC-conjugated anti CD 11b antibody (eBiosciences, San Diego, CA.) After washing, the stained cells were analyzed by flow cytometry (BD Biosciences, Franklin Lakes, NJ) and the percentage of CD11b positive cells calculated with FlowJo 6.3 software. Top and middle panel, a representative histograph of CD11b stained U937 cells stably transfected with pcDNA3 (top) and MOZ-TIF2 (middle). Y-axis, % cell number and X-axis, APC stain intensity. The horizontal line scale represents the range of CD11b positive cells. The dashed and solid lines represent basal and RA treated U937 cells, respectively. The number above the line scale indicates percentage of CD11b positive cells after treatment with RA. Bottom panel, percentage of CD11b positive U937 cells stably transfected with control (pcDNA3), MOZ-TIF2 (MT2), MT2 del 82–892, and MT2 del CID. The experiment was conducted in triplicates with the open bars representing the basal level of CD11b positive cells and the dark bars representing the level of CD11b after RA treatment. Double stars represent a significant difference at P < 0.01 by a two tailed Student T- test compared to the RA treated U937 cells with MT2-2. The percentage inhibition cited in the text was calculated from the amount of RNA or protein expressed in MOZ-TIF2 expressing cells compared to that in pcDNA-transfected control cells in presence of RA subtracted from 100%.

To support our findings with the reporter systems, we examined the inhibition of endogenous RA response genes in U937 cells with stable expression of MOZ-TIF2. The expression level of MOZ-TIF2 RNA in two established clones, MT2-1 and MT2-1, was similar (MT2-1) to or lower (MT2-2) than that seen in the patient's leukemic blasts (Figure 9A). Expression of two well-known RA response genes, C/EBPβ (Figure 9B and 9C) and CD11b (Figure 9D and 9E) were examined in the two U937 clones at the RNA (Figure 9C and 9D) and protein level (Figure 9B and 9C). Induction of C/EBPβ RNA by RA was inhibited by 86% in clone MT2-1 and 40% in clone MT2-2 (Figure 9C). and C/EBPβ protein expression after RA treatment was reduced by 90% in MT2-1 and 54% in MT2-2 (Figure 9B), respectively, compared to that of control cells. Similarly, induction of CD11b RNA by RA was decreased by 60% in both MT2-1 and MT2-2 clones (Figure 9D). A flow cytometry analysis showed that the number of CD11b positive cells in the MT2-2 clone of MOZ-TIF2 expressing U937 cells was less than 50 % of control cells (Figure 9E). MOZ-TIF2 with deletions either of the MOZ portion or CID domain increased the number of CD 11b positive cells to 80% of control cells.

## Discussion

Previously, MOZ-TIF2 has been shown using reporter systems to repress both RA and PPAR gamma receptor mediated transcription in U937 cells and p53 mediated transcription in p53 null cell lines [41]. In the investigations reported here, we used ER- and AR-mediated transcription reporter systems as models to study the effect of MOZ-TIF2 on nuclear receptor signaling in CV-1 and HEK293 cells, lines that have been widely used for the investigation of nuclear receptor signaling. We found that the MOZ-TIF2 fusion inhibited both ER and AR-mediated transcription compared both to the pcDNA3 vector alone and to MOZ and TIF2. The transcription inhibition by MOZ-TIF2 in ER mediated transcription occurred in both basal and estradiol-stimulation conditions. In the AR-mediated transcription system, MOZ-TIF2 exerted a strong suppression only of DHT induced transcription. The differences in the effect of MOZ-TIF2 on basal transcription in the ER and AR reporter systems may result either from MOZ-TIF2 exerting a ligand independent effect on the ERE binding element or promoter specific effects similar to those reported for other hormone receptors [46]. We also observed that in the AR-mediated transcription system TIF2 decreased the response to DHT though to a far lesser extent than MOZ-TIF2. The effect of TIF2 again suggests promoter specific effects which may be related to the previously described interactions between LXXLL motifs of TIF2 with the ligand-binding domain of the androgen receptor that allowed for negative regulation [47]. Some support for this explanation comes from the observation that TIF2 inhibits DHT stimulation of the PSA promoters though to a lesser degree than MOZ-TIF2. In addition, we demonstrated that expression of MOZ-TIF2 in U937 cells at levels similar to that of patient's leukemic blasts inhibited the response of endogenous genes, C/EBPβ and CD11b to RA, suggesting that MOZ-TIF2 could decrease nuclear receptor-mediated transcription *in vivo*.

To explore the universal inhibition of MOZ-TIF2 in nuclear receptor-mediated transcription, we tested the effect of MOZ-TIF2 on ER mediated transcription in K562 and HEK 293 cells. Interestingly, the inhibition by MOZ-TIF2 of basal and ligand-dependent transcription in an ER reporter system was not as great as observed in CV-1 cells, indicating that the repression by MOZ-TIF2 of nuclear receptor-mediated transcription may be cell-dependent. Cell specific effects are supported by the observation that MOZ-TIF2 repressed a p53-responsive reporter plasmid in CV-1 and HEK293 cells to a far lesser extent (data not shown) than has been reported in SaOs2 cells [41].

The MYST domain in MOZ has HAT activity and the domain is retained in the MOZ-TIF2 fusion. In our systems, a mutation in the acetyl-CoA binding site (G657D) of the MYST domain, which is known to abolish HAT activity [40], did not abrogate MOZ-TIF2-mediated repression of ER-mediated transcription and had only a slight effect on AR-mediated transcription (data not shown). These results imply that HAT activity of the MOZ portion has no functional significance in nuclear receptor mediated transcription by MOZ-TIF2 fusion protein. The non-essential role of HAT activity in the MOZ fusion protein has been observed previously as the abolishment of HAT activity in the MOZ-CBP fusion protein did not significantly change AML1-mediated transcription activity and the same mutation in MOZ-TIF2 was not required for transformation of myeloid cells while C2HC nucleosome recognition motif was essential for MOZ-TIF2 transformation [17,40].

In nuclear receptor-mediated transcription, protein acetylation may be provided by recruitment of the coactivators, CBP/p300 via the p160 SRC family [48,49]. CBP/p300 are universal transcription coactivators with HAT activity [50,51] that participate in multiple transcriptional events mediated by viral oncoproteins, hematopoiesis-related transcription factors, tumor suppressors, and nuclear receptors through the regulation of histone acetylation and interaction with the basal transcriptional machinery [52-55]. CBP/p300 also contributes to the acetylation of non-histone proteins, such as Rb, E2F, and P53 and regulates cell growth and differentiation. Both deficient CBP/p300 and mutated CBP/p300 are relevant to the hematopoietic malignancies and solid tumors [56-59]. In the MOZ-TIF2 fusion, CID, a CBP interacting domain in TIF2 portion, is retained and this domain is required for inhibition of RA and PPRAγ receptor-mediated transcription and for leukemogenesis [41]. We demonstrated that the deletion of this domain in MOZ-TIF2 removed the repression of ER and AR-mediated transcription and partially reversed the response of endogenous CD11b to RA. Furthermore, the mutations in two leucine-rich repeats of the CID showed a similar effect as the deletion. In contrast, the deletion of CID from TIF2 suppressed the ER and AR-mediated transcription suggesting that the binding of MOZ-TIF2 to CBP/p300 played a direct role in inhibition of ER and AR-mediated transcription by MOZ-TIF2. The expression level of CBP in cells has been reported as an important factor in the inhibition of p53-mediated transcription by MOZ-TIF2 [41]. However, in the nuclear receptor-mediated transcription model increased expression of CBP did not alter the inhibition of ER-mediated transcription by MOZ-TIF2. In contrast, co-expressed wild type TIF2 interfered with MOZ-TIF2 inhibition suggesting that TIF2 may be an important competitor and cellular levels of TIF2 could modify inhibition of nuclear receptor mediated transcription.

By co-immunoprecipitation we demonstrated that ER, CBP, and MOZ-TIF2 were in same protein complex. It is known that in nuclear receptor-mediated transcription TIF2 recruits CBP/P300 via CID [60]. Therefore, the interaction between CBP and MOZ-TIF2 could be explained by the binding of the CID moiety in the TIF2 portion of the fusion protein. However, we found that CBP was also co-precipitated by wild type MOZ, which suggested that the MOZ portion of MOZ-TIF2 could interact with CBP directly. In support of the participation of MOZ, the physical interaction between the MOZ portion (amino acids 1–759) of MOZ-TIF2 and the C-terminal of CBP (amino acids 1680 to 2441) was shown in GST pull-down experiments. The mapping of the CBP binding region in the MOZ N-terminus suggests multiple CBP binding sites. Analysis of the MOZ protein sequence shows a CBP binding sequence, FX(D/E)XXXL, is located in the MYST domain at amino acids 600–606. In addition, four binding consensus-like motifs, XX (D/E) XXXL, are in the PHD domain of MOZ (amino acids 1–253) and three XX (D/E) XXXL motifs are located in the MYST domain. It has been shown that any single substitution in FX (D/E) XXXL was not able to block the binding of E1A to CBP [45]. The MOZ portion binding sites in CBP were in the CH3 domain which contains a transcriptional adapter motif (TRAM) (amino acids 1811–1822) which binds competitively to p53, E1A, E2F, and overlaps with the binding sites for mdm2, myoD, and P/CAF. The analysis of solution structure supports the binding ability of this domain [61]. However, our results suggest that there is/are some CBP binding site(s) even beyond the PHD and MYST domains because a MOZ-TIF2 fragment with the deletion of both domains still bound to CBP. Interestingly, each CBP binding site in MOZ portion may be of similar importance in the suppression of nuclear hormone-stimulated transcription by MOZ-TIF2.

Co-activation of ER and AR by p160 SRC family members is through two sites of interaction. One interaction occurs between the NID of the p160 proteins and the AF2 domain of ER and AR. The other interaction occurs between the C-terminus of p160 proteins and the AF1 domain in N-terminus of ER and AR and within AR the later interaction is stronger than former [62-66]. In the MOZ-TIF2 protein, the NID of TIF2 has been eliminated by the chromosomal translocation. Loss of this domain will change the interaction of MOZ-TIF2 with ER and AR. In our study, although ER was co-immunoprecipated with MOZ-TIF2 the ER level was almost half that which was pulled down by wild type TIF2. It seems that MOZ-TIF2 may participate in the transcriptional complex of ER in a way different from wild type TIF2. There are two alternative ways for MOZ-TIF2 to interact with ER: MOZ-TIF2 could interact with the AF1 domain of ER or MOZ-TIF2 could interact through other complex members such as CBP which has been shown to bind directly to ER [67,68].

## Conclusion

Our work conclusively suggests that MOZ-TIF2 as a bidentate CBP binding protein competes with wild type TIF2 in ER and AR mediated transcription. The aberrant binding to ER or AR and CBP by MOZ-TIF2 disorders the complex for receptor signaling and may bring about abnormal acetylation of histone and non-histone proteins or cause other aberrant modifications, which finally lead to the inhibition of transcription activation by liganded ER and AR. In addition, the MOZ portion of the fusion protein not only determines the localization of MOZ-TIF2 within the cell but also contributes to the inhibition by MOZ-TIF2 of nuclear receptor activation.

## Methods

### Plasmids

The MOZ-TIF2 fusion cDNA was constructed by the joining of the PCR-created fusion fragments to the MOZ partner at a Hind3 site and the TIF2 partner at a Sac1 site in pBluescript KS phagemid vector. The fusion cDNA was subsequently subcloned into the pcDNA 3.1A (Invitrogen, Carlsbad, CA), pEGFP (BD Biosciences Clontech, Palo Alto CA), pGEX (Amersham Pharmacia Biotech, Piscataway, NJ), and pET-30 (Novagen, Madison WI). The luciferase reporter plasmid containing two ER binding elements plus a TK minimal promoter (Vit TKSL) was a kind gift from Dr. James Mathis. The luciferase reporter plasmid driven by the long promoter/enhancer (PL) carrying the sequence between -6480 and +12 nucleotides of PSA (prostate specific antigen) or driven by the core promoter (PS) from -648 to +12 nucleotides of PSA was a gift from Dr. Stephen P. Balk [69,70].

### RNA isolation and RT-PCR

RNA was isolated from transduced or transfected cells and leukemic blasts of patients with TRI Reagent (Molecular Research Center, Inc., Cincinnati OH). PCR primers were designed to yield amplicons ranging in length from 270 to 776 bp with Tm between 52.5 and 60.5°C, with most of the primer sets having an optimum Tm of 56–57°C (Table 1). One microgram of RNA was reversed transcribed with Superscript™ II, RNase H- Reverse RT-PCR as previously described [71]. Briefly, the RT mixture was diluted 10–1000 fold in water so that amplicons of different abundances could be amplified in parallel with the same number of cycles. Based on the abundance of each amplicon in preliminary analyses, target sequences in 1 μL of the appropriate dilution of the cDNA reaction were amplified in 10 μL reactions containing 0.25 units of Taq, 2 pmol each of the sense and antisense primers in PCR buffer, 62.5 μM of each dNTP, and 0.05 μL [α-^32^P]dCTP. Control PCR reactions using no added DNA template and using no RT were done as sets after the cDNA amplifications were complete. Following electrophoresis through 1.5% Tris-Borate-EDTA (TBE) agarose gels, the amplicons were transferred by electroblotting to GeneScreen Plus nylon membranes (Dupont, Boston, MA), the images detected on a Molecular Dynamics PhosphorImager, and analyzed by volume integration with the ImageQuant software. The expression levels for each of the genes were normalized to the GAPDH expression. In some experiments, regular PCR or real-time PCR was conducted without [α-^32^P]dCTP.

**Table 1:** The primer sets applied in PCR

Gene	bp	upstream primer	downstream primer
MOZ	481	CGT CGC TAC AGT GAG GGT GA	G TTT TCG CAA AAG AGA TAC TGG CT
MOZ	577	CGT CGC TAC AGT GAG GGT GA	A GTG GAT TGG TTT GCG GCT C
p300	361	GA GCA CCC GTT GGA CTT GGA	GA GGG CAC ACT GGC ATT TTC A
CBP	399	GAG GTT TTT GTC CGA GTG GTG G	TGG GTG GCA ATG GAA GAT GTA A
PCAF	309	CA AAC GCA GGG AGC AGC AGT	T GTT TGG TTT CTG GTT GAG GGA
PCAF	384	CA AAC GCA GGG AGC AGC AGT	T GTA TTC TTT TGG CAT TCG GGG
SRC1	300	GG CAG CAA GGA GCG ATA GGA	G GGT TCC ATC TGC TTC TGT TTT G
TIF2	638/428	CG GTG AGC CCC AAG AAG AAA	CCG AGA AGC ACT GTT ACC AAT CAT
TIF2	270/60	GGA GCC CAG AAA ACA GCA CT	CCG AGA AGC ACT GTT ACC AAT CAT
TIF2	578/368	GGA GCC CAG AAA ACA GCA CT	GC AAA AGA CGC CTG GTC TAT
MOZ/TIF2	776	CGT CGC TAC AGT GAG GGT GA	GC AAA AGA CGC CTG GTC TAT
MOZ/TIF2	468	CGT CGC TAC AGT GAG GGT GA	CCG AGA AGC ACT GTT ACC AAT CAT
NCOA3	395	C CAA GCA GCA GCA TCT AAC CAA C	G TCC CTG AAG ATG AAA GCC TCC T
CD11b	73	AGT TGC CGA ATT GCA TCG A	GGC GTT CCC ACC AGA GAG A
C/EBP b	225	CCT CGC AGG TCA AGA GCA A	ACA AGT TCC GCA GGG TGC T

Gene, gene symbol. bp, the PCR fragment length in base pairs.

### Mutation and deletion

Both point and deletion mutations in the MOZ-TIF2 fusion cDNA were constructed with QuikChange^® ^Site-Directed Mutagenesis Kit (Stratagene, La Jolla CA). The primers used for the deletion and mutation are listed in Table 2. All mutations were verified by sequencing.

**Table 2:** The primers used for the deletion and mutation

Del 83–180	5'-GATCCTGATAATCCTGGGCGAATAGCACTTAGTTGTGAGTCTCTTTCCTGTTTACCTCCA
Del 41–738	5'-AGGATATGCAATGCTGTGTCTTCATCCCATGACTTCCGTAGTGACCAATTTGTGATTATC
Del 193–346	5'-GAGTCTCTTTCCTGTTTACCTCCAGTGTCCACGGTATCAAAAGGTCCCTTCAGCAAAGTT
Del 486–764	5'-ATGACTGAGAAAGATATGGAATTATTTCGTCCTGTAGATGTAGATCCAGAATGTTTGCGC
Del 82–892	5-GATCCTGATAATCCTGGGCGAATAGCACTTTCAGCTCCTCAGGAACAATATGGAGAATG
Del 14–1107	5'-CTCGCAAACCCGCTTTATACTGAGTGGATTGAAGAAGATGAAGAGTCAGATGATGCTGAT
Del 909–1107	5'-ACCCAGGAACAATACACTGAAAGTGAAGAAGAAGAAGATGAAGAGTCAGATGATGCTGAT
Del 1182–1382	5'-GCAGCCAGCCTGGCCAAAGACAGACGAACATCATGCTGGAGCAGA
Mut 1241–1244	5'-GCCATTTGGCAGTTCTCCAGCGGCCGCAGCATGTCCACATCCTGCAGC
Mut 1259–1260	5'-CCGAGTGATGAGGGAGCTGCCGCAGACCAGGCCTATCTGGCCTTGCGG

The left column indicates the region of deletion or mutation of the MOZ protein.

### Cell culture and transfection

HEK293 and CV-1 cells were grown in DMEM (Mediatech Cellgro, Herndon VA) containing 10% fetal bovine serum (FBS). K562 AND U937 cells were grown in RPMI1640 with 10% FBS. Transfections of HEK293 and CV-1 cells were done with Lipofectamine Plus (Invitrogen, Carlsbad CA) and of K562 cells with FuGENE 6 transfection reagent (Roche Applied Science, Indianapolis IN). U937 cells were transfected by electroporation. Transiently transfected cells were harvested 24–48 hours after transfection as indicated for the specific studies. Stable transfected cells were selected with G418 for 2–3 weeks.

### Subcellular location by fluorescent microscopy and immunofluorescence staining

To determine the subcellular location of MOZ-TIF2, a GFP fusion of MOZ-TIF2 was expressed in HEK293, K562, and CV1 cells by transient transfection. After 24 hours the cells were fixed in 1% paraformaldehyde and examined by epifluorescence microscopy. To examine localization of the MOZ-TIF2 protein and protein products of various MOZ-TIF mutations in living cells, cells were cultured in 6-well plates. After 24 hours of transfection, cells were stained with DRAQ5™ (AXXORA, LLC, San Diego, CA) and images collected on a laser scanning confocal microscope (Bio-Rad Laser Scanning System Radiance 2000/Nikon Eclipse TE300 microscope) with LaserSharp 2000 software (Bio-Rad, Hercules, CA). To observe co-localization between endogenous MOZ and CBP, HEK293 and Hela cells were fixed in 1% paraformaldehyde, blocked with 3% bovine serum albumin in PBS, and incubated with antibodies (Santa Cruz Biotechnology, Inc., Santa Cruz, CA) against MOZ (N-19, sc-5713) at 1:100 and CBP (C-1, sc-7300) at 1:200. Nuclei were counterstained with TO-PRO^®^-3 iodide (In Vitrogen-Molecular Probes, Carisbad, CA) and images collected as above.

### Luciferase assay

CV1 and HEK 293 cells were transfected by Lipofectamine Plus in 24-well plates with 350 to 400 ng of luciferase reporter plasmids, 100 ng of expression plasmids of MOZ, MOZ-TIF2, TIF2, MOZ-TIF2 mutants, and 50 ng of estrogen or androgen receptor expression vector. After 36 hours of induction with 50 nM of estradiol (E) or 5α-dihydrotestosterone (DHT) in medium containing 10% dextran-charcoal-stripped fetal calf serum, cells were lysed with Cell Culture Lysis Reagent (Promega, Madison WI) and the luciferase assay was performed in a Monolight^® ^2040 luciferase luminometer by adding 10 μl of cell lysate to 100 μl of reaction mix consisting of 1 × salt buffer with pH 7.8 (20 mM of Tricine, 1.07 mM (MgCO_3_)_4_Mg (OH)_2· _5H_2_O, 2.67 mM MgSO_4_, 0.2 mM EDTA, 530 μM ATP, 33.3 μM DTT, 270 μM coenzyme A, and 470 μM potassium luciferin. The luciferase activity was standardized for transfection efficiency with β-galactosidase activity. All experiments were performed at least in quadruplicate and repeated at least twice.

### Co-immunoprecipitation and immunoblotting

HEK293 cells were transfected either with EGFP or a His-tag fusion of MOZ, MOZ-TIF2, or TIF2. In some experiments, a vector expressing the human estrogen receptor was co-transfected. Cell lysates were prepared with individual homogenizers in lysis buffer (50 mM NaCl, 5 mM KCl, 1 mM EDTA, 20 mM HEPES, pH 7.6, 10% glycerol, and protease inhibitor cocktails (Roche Applied Science, Indianapolis IN)). Immunoprecipitation was conducted with antibodies against His-tag or EGFP. Briefly, 2 μg of agarose-conjugated anti-His-tag (Santa Cruz Biotechnology, Santa Cruz CA) or anti-GFP antibody (BD Biosciences, Palo Alto CA) bound to protein A/G-agarose (Santa Cruz Biotechnology, Santa Cruz CA) was added to about 500 μg protein of cell lysate, incubated at 4°C overnight with rotation, the precipitate collected by centrifugation and washed with phosphate buffered saline (PBS) containing 0.5 % NP-40. After the final wash, the pellet was separated by SDS-PAGE and the western analysis conducted with antibodies against CBP and estrogen receptor. In some experiments, RIPA buffer was used to extract whole cell protein for western analysis.

### GST pull down assay

The GST fusion proteins were expressed with pGEX constructs containing designated cDNA fragments in BL21-CodonPlus^®^(DE3)-RIL cells (Stratagene, La Jolla CA). The expressed GST fusion protein was purified with the GST Purification Module (Amersham Pharmacia Biotech, England), fractionated by SDS-PAGE, and proteins detected by staining with Coomassie Blue. To perform the GST pull down affinity assay, [^35^S] Met-labeled proteins were produced with the Single Tube Protein^® ^System 3 (Novagen, Madison WI) from pET 30 vectors containing designated cDNA fragments. The binding reaction was conducted with 5 μl of in vitro-translated protein and 3–5 μg of GST or GST fusion protein bound to Sepharose 4B beads in 200 μl binding buffer (50 Mm Tris-HCl, pH 8.0, 100 mM NaCl, 0.3 mM DTT, 10 mM MgCl2, 10% glycerol, 0.1% NP40). The reaction was performed at 4°C for 1 hour followed by five washes of the beads with binding buffer, separation of bound proteins by SDS PAGE followed by autoradiography. In some experiments, non-isotope labeled X-press-tagged proteins were used and proteins pulled down by GST-fusion were examined by anti X-press antibody (Invitrogen Co., Carisbad, CA).

## Competing interests

The author(s) declare that they have no competing interest.

## Authors' contributions

HY participated in development of concept and design, performed experiments, analyzed data, draft manuscript. JG contributed to experiment design, data interpretation, manuscript revision, and final approval. KLB contributed to idea development, experiment design and performance, and data interpretation. All authors read and approved the manuscript.
